# Supplementary material for: Enhanced optical path and electron diffusion length enable high-efficiency perovskite tandems
Source: Nat Commun. 2020 Mar 9;11:1257. doi: 10.1038/s41467-020-15077-3 (PMC7062737; doi:10.1038/s41467-020-15077-3)
Supplement: Supplementary file 1 — Supplementary Information [file 41467_2020_15077_MOESM1_ESM.pdf]

# Enhanced Optical Path and Electron Diffusion Length Enable High-Efficiency Perovskite Tandems

Bin Chen[1†], Se-Woong Baek[1†], Yi Hou[1†], Erkan Aydin[2], Michele De Bastiani[2], Benjamin Scheffel[1], Andrew Proppe[1], Ziru Huang[1], Mingyang Wei[1], Ya-Kun Wang[1], Eui-Hyuk Jung[1], Thomas G. Allen[2], Emmanuel Van Kerschaver[2], F. Pelayo García de Arquer[1], Makhsud I. Saidaminov[1], Sjoerd Hoogland[1], Stefaan De Wolf[2], and Edward H. Sargent[1]*

[1]Department of Electrical and Computer Engineering, University of Toronto, 35 St George Street, Toronto, Ontario M5S 1A4, Canada.

[2]King Abdullah University of Science and Technology (KAUST), KAUST Solar Center (KSC), Physical Sciences and Engineering Division (PSE), Thuwal, 23955-6900 Kingdom of Saudi Arabia

†These authors contributed equally to this work
*Correspondence to: ted.sargent@utoronto.ca

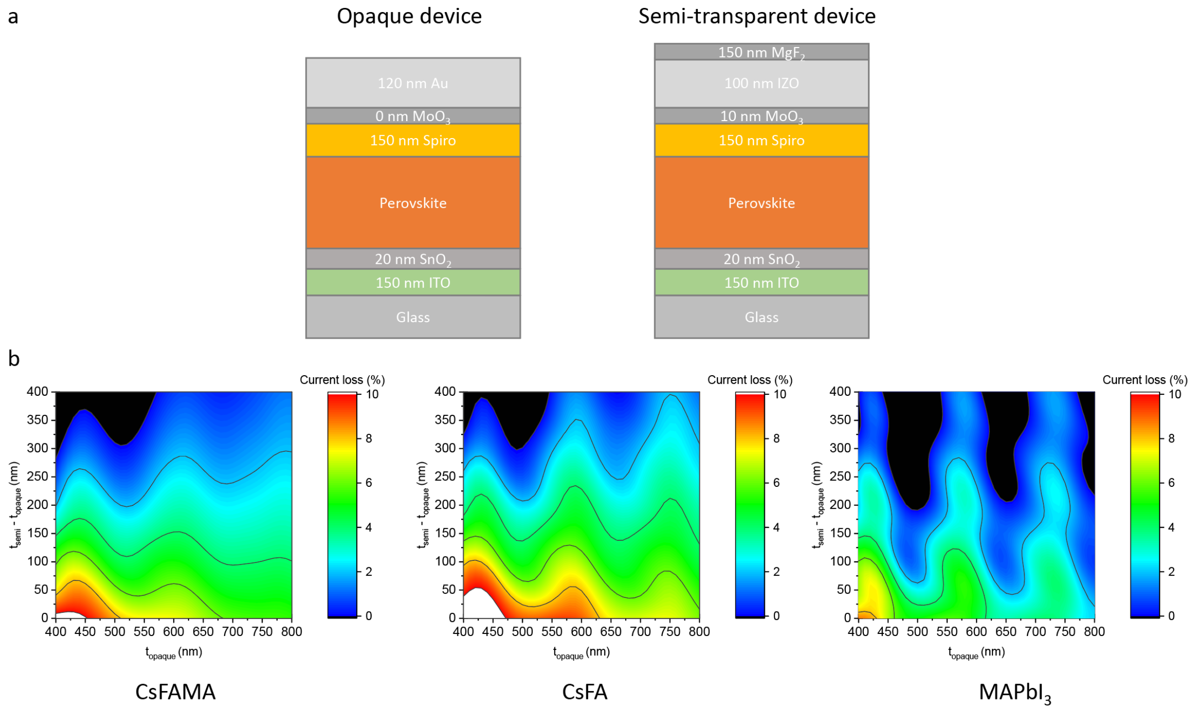


**Supplementary Figure 1| a,** structure of opaque and semi-transparent perovskite devices used for transfer matrix calculation and experiment. **b,** Transfer matrix calculation of extra active layer thickness in semi-transparent device required to compensate for the transmitted photon loss for CsFAMA, CsFA and $MAPbI_3$. Refractive index of layers (except CsFAMA perovskite) used in calculation were obtained from https://www.pvlighthouse.com.au/refractive-index-library.

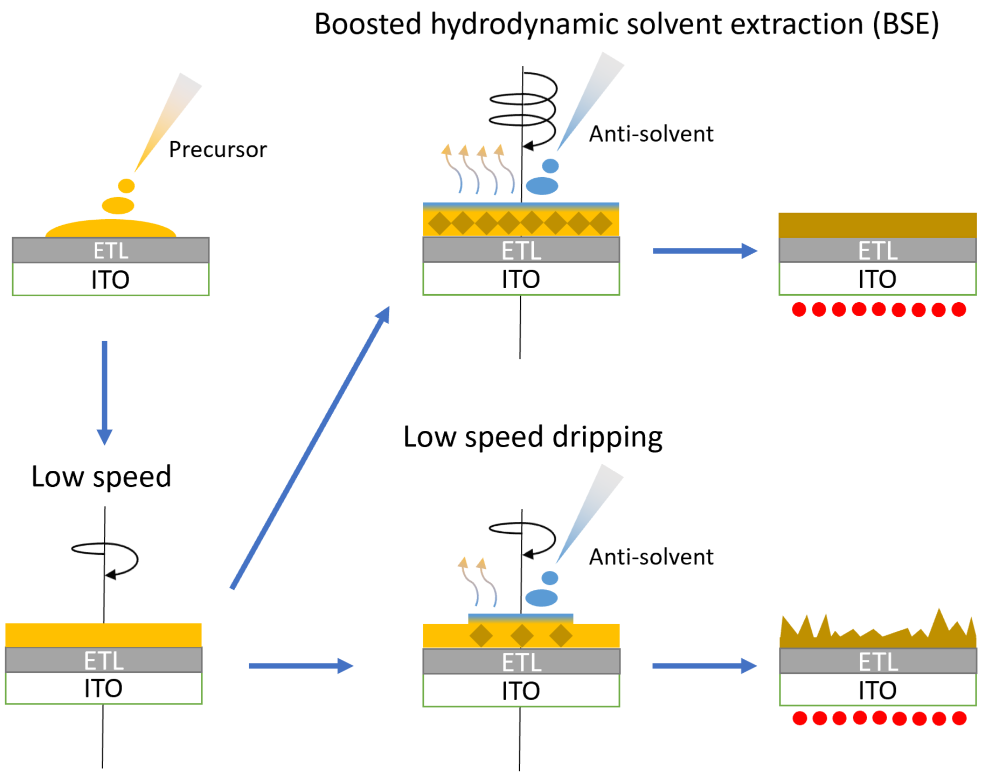


**Supplementary Figure 2**| Schematic of boosted solvent extraction (BSE) and normal spin coating process.

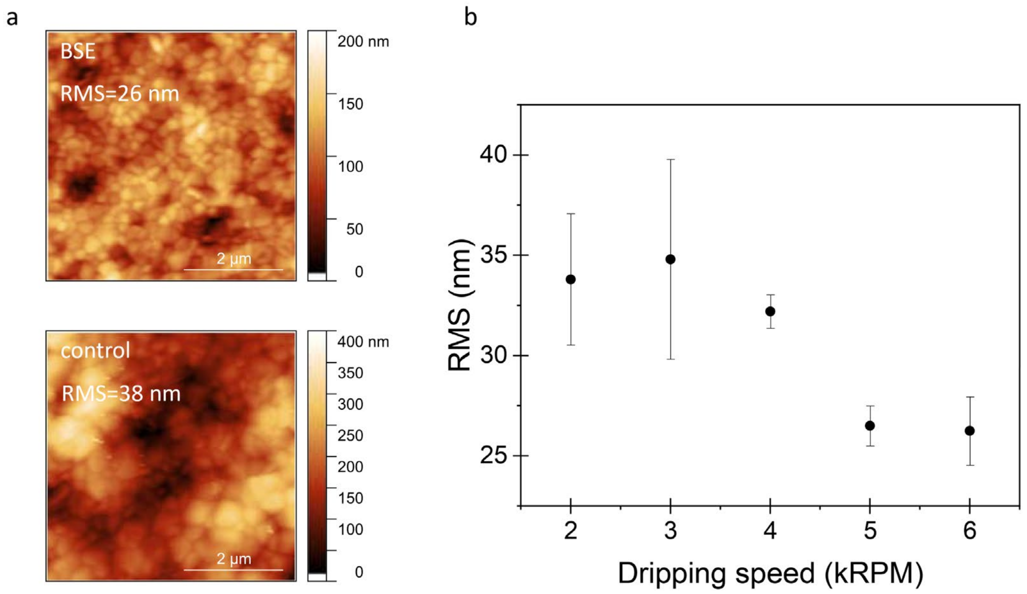


**Supplementary Figure 3| a,** AFM images of BSE and non-BSE film. **b,** Surface roughness vs. spin speed of anti-solvent dripping.

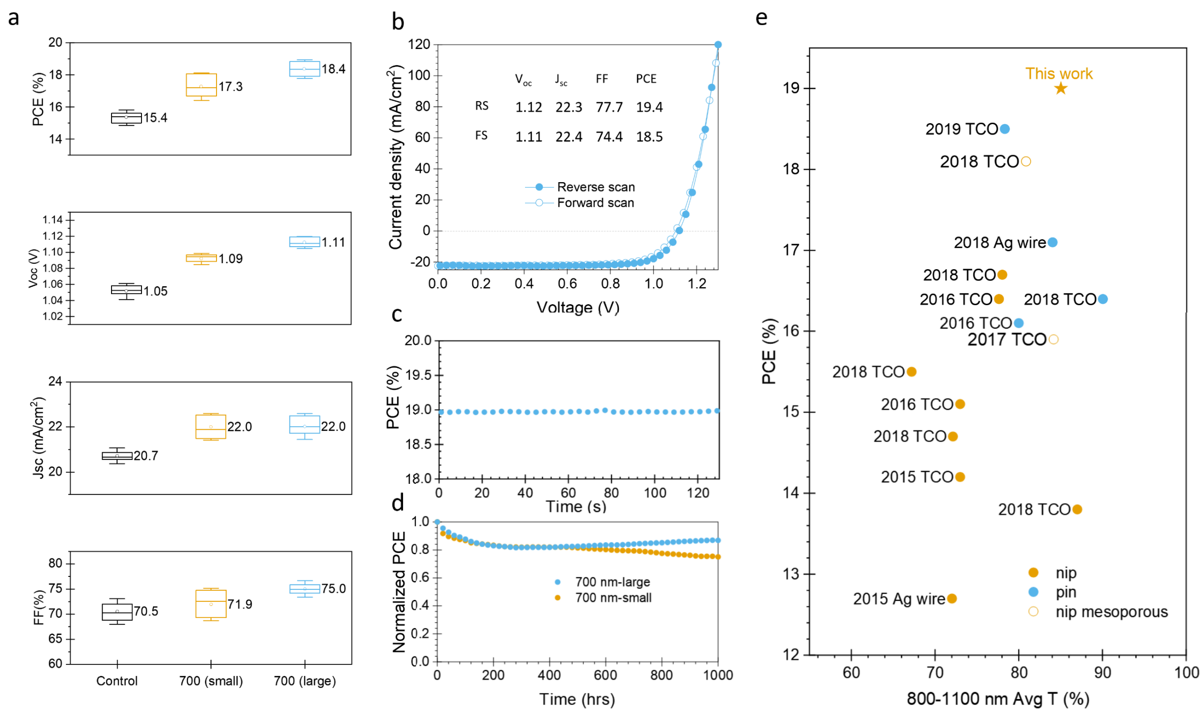


**Supplementary Figure 4| a,** PV parameters for control devices prepared by non-BSE and by BSE (700 nm-small) and by BSE+Urea (700 nm-large), based on 30 devices each. **b,** Long-term MPP tracking for both 700 nm-small and 700 nm-large devices. **c,** Forward and reverse scan of champion 700 nm-large perovskite device. Inset: Shor-term maximum power point tracking showing the stabilized PCE of champion device. **d,** a summary of performance of semi-transparent perovskite devices in literature.

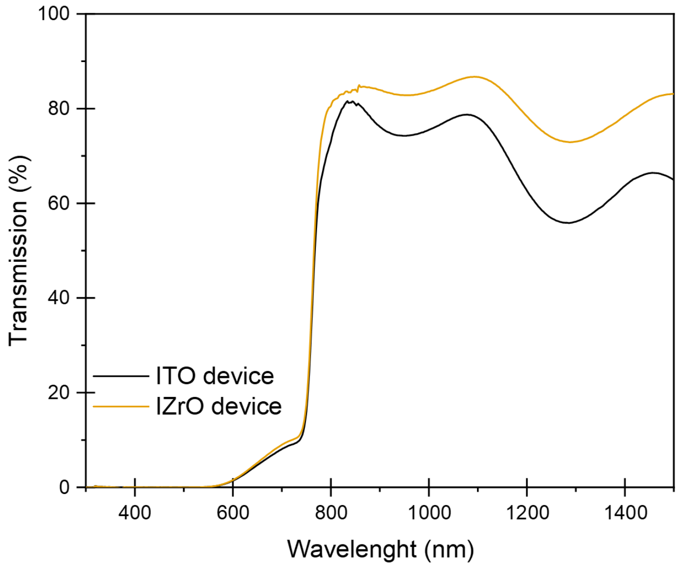


**Supplementary Figure 5|** Optical transmittance of the semi-transparent perovskite devices with commercial ITO substrate vs. Zr doped $In_2O_3$ (IZrO) substrate.

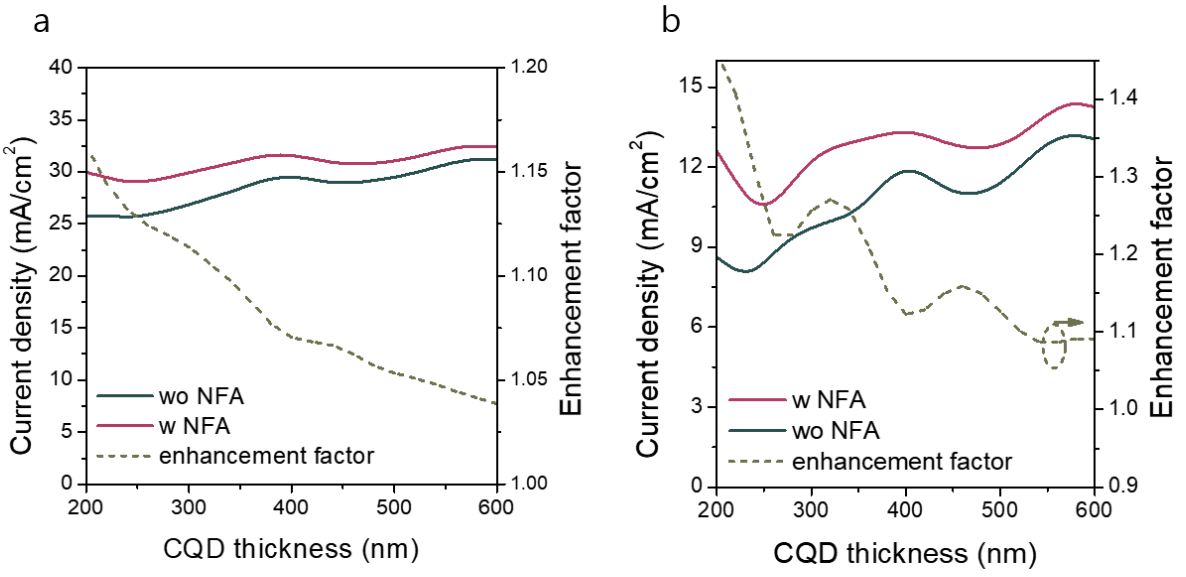


**Supplementary Figure 6|** Calculated current density of CQD cells with (purple) and without (green) NFA for **a,** full spectrum A1.5 illumination and **b,** after 760 nm filter cutoff illumination. Dot lines in panel **a-b** denote $J_{sc}$ enhancement in the presence of NFA.

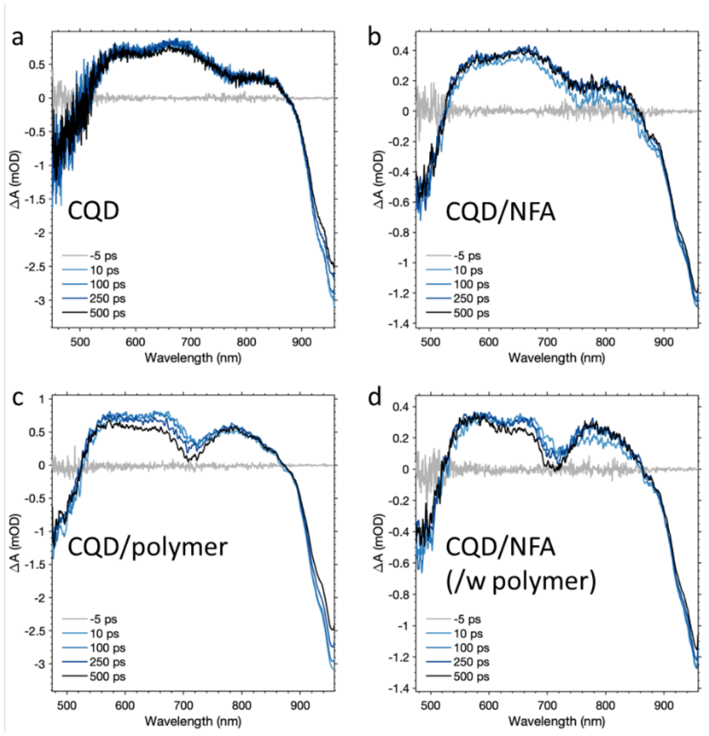


**Supplementary Figure 7|** Transient absorption (TA) spectra of **a,** CQD, **b,** CQD/NFA, **c,** CQD/polymer and **d,** CQD/NFA (with polymer). The excitation wavelength is 950nm.

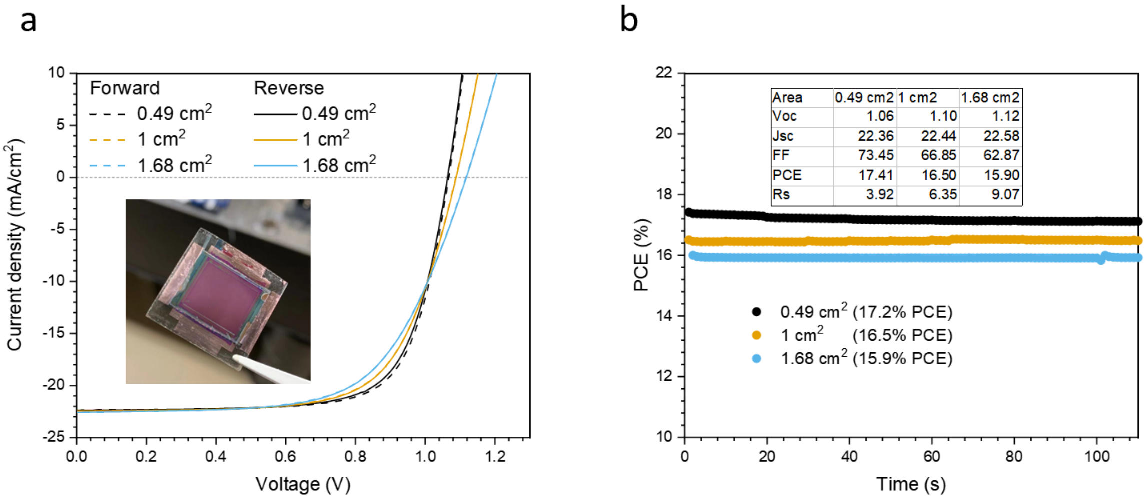


**Supplementary Figure 8| a**, *JV* curves and **b**, stabilized power output of 1.95 cm$^2$ devices with various mask sizes. Inset is the image of the corresponding device.

**Supplementary Table 1|** A summary of perovskite-silicon tandem efficiency in literature.

| Type | PCE (%) | PSC cell area ($cm^2$) | PSC cell filter area ($cm^2$) | Reference |
|---|---|---|---|---|
| 4T | 28.2 | 0.049 | 4 | This work |
| | 25.7 | 1 | 4 | This work |
| | 17 | 0.39 | 0.39 | 1 |
| | 19.8 | 0.0919 | 0.0919 | 2 |
| | 21.4 | 0.09 | 0.09 | 3 |
| | 25.2 | 0.25 | 0.25 | 4 |
| | 25.3 | 0.13 | 4 | 5 |
| | 25.5 | 0.09 | 0.09 | 6 |
| | 26.1 | NA | NA | 7 |
| | 26.2 | 0.16 | 1 $in^2$ | 8 |
| | 26.4 | 0.16 | 4.84 | 9 |
| | 26.9 | 0.104 | 0.104 | 10 |
| | 27.1 | 0.13 | 0.13 | 11 |
| 2T | 13.7 | 1 | NA | 12 |
| | 16.4 | 0.25 | NA | 13 |
| | 18.1 | 0.16 | NA | 14 |
| | 20.5 | 1.43 | NA | 4 |
| | 20.5 | 4 | NA | 15 |
| | 20.6 | 0.03 | NA | 16 |
| | 21.2 | 0.17 | NA | 17 |
| | 21.8 | 16 | NA | 18 |
| | 22.2 | 0.06 | NA | 19 |
| | 22.5 | 1 | NA | 20 |
| | 22.7 | 1 | NA | 21 |
| | 23.6 | 1 | NA | 22 |
| | 24.1 | 1 | NA | 23 |
| | 25 | 1 | NA | 24 |
| | 25.1 | 1.42 | NA | 25 |
| | 25.2 | 1.09 | NA | 26 |
| | 25.2 | 1.42 | NA | 27 |
| | 25.4 | NA | NA | 28 |
| | 25.5 | 0.81 | NA | 29 |
| | 26.0 | 0.77 | NA | 30 |

**Supplementary References**


1. Bailie, C. D. *et al.* Semi-transparent perovskite solar cells for tandems with silicon and CIGS. *Energy Environ. Sci.* **8**, 956–963 (2015).
2. McMeekin, D. P. *et al.* A mixed-cation lead mixed-halide perovskite absorber for tandem solar cells. *Science* **351**, 151–155 (2016).
3. Kanda, H. *et al.* Effect of Silicon Surface for Perovskite/Silicon Tandem Solar Cells: Flat or Textured? *ACS Appl. Mater. Interfaces* **10**, 35016–35024 (2018).
4. Werner, J. *et al.* Efficient Near-Infrared-Transparent Perovskite Solar Cells Enabling Direct Comparison of 4-Terminal and Monolithic Perovskite/Silicon Tandem Cells. *ACS Energy Lett.* **1**, 474–480 (2016).
5. Jaysankar, M. *et al.* Perovskite–silicon tandem solar modules with optimised light harvesting. *Energy Environ. Sci.* **11**, 1489–1498 (2018).
6. Dewi, H. A. *et al.* Highly Efficient Semitransparent Perovskite Solar Cells for Four Terminal Perovskite-Silicon Tandems. *ACS Appl. Mater. Interfaces* **11**, 34178–34187 (2019).
7. Zhang, D. *et al.* Highly near-infrared-transparent perovskite solar cells and their application in high-efficiency 4-terminal perovskite/c-Si tandems. in *2018 IEEE 7th World Conference on Photovoltaic Energy Conversion (WCPEC) (A Joint Conference of 45th IEEE PVSC, 28th PVSEC & 34th EU PVSEC)* 3575–3577 (IEEE, 2018). doi:10.1109/PVSC.2018.8547936.
8. Aydin, E. *et al.* Zr-Doped Indium Oxide (IZRO) Transparent Electrodes for Perovskite-Based Tandem Solar Cells. *Adv. Funct. Mater.* **29**, 1901741 (2019).
9. Duong, T. *et al.* Rubidium Multication Perovskite with Optimized Bandgap for Perovskite-Silicon Tandem with over 26% Efficiency. *Adv. Energy Mater.* **7**, 1700228 (2017).
10. Quiroz, C. O. R. *et al.* Balancing electrical and optical losses for efficient 4-terminal Si–perovskite solar cells with solution processed percolation electrodes. *J. Mater. Chem. A* **6**, 3583–3592 (2018).
11. Jaysankar, M. *et al.* Minimizing Voltage Loss in Wide-Bandgap Perovskites for Tandem Solar Cells. *ACS Energy Lett.* **4**, 259–264 (2019).

12. Mailoa, J. P. *et al.* A 2-terminal perovskite/silicon multijunction solar cell enabled by a silicon tunnel junction. *Appl. Phys. Lett.* **106**, 121105 (2015).

13. Werner, J. *et al.* Zinc tin oxide as high-temperature stable recombination layer for mesoscopic perovskite/silicon monolithic tandem solar cells. *Appl. Phys. Lett.* **109**, 233902 (2016).

14. Albrecht, S. *et al.* Monolithic perovskite/silicon-heterojunction tandem solar cells processed at low temperature. *Energy Environ. Sci.* **9**, 81–88 (2016).

15. Zheng, J. *et al.* Large area efficient interface layer free monolithic perovskite/homo-junction-silicon tandem solar cell with over 20% efficiency. *Energy Environ. Sci.* **11**, 2432–2443 (2018).

16. Fan, R. *et al.* Toward Full Solution Processed Perovskite/Si Monolithic Tandem Solar Device With PCE Exceeding 20%. *Sol. RRL* **1**, 1700149 (2017).

17. Werner, J. *et al.* Efficient Monolithic Perovskite/Silicon Tandem Solar Cell with Cell Area >1 $cm^2$. *J. Phys. Chem. Lett.* **7**, 161–166 (2016).

18. Zheng, J. *et al.* 21.8% Efficient Monolithic Perovskite/Homo-Junction-Silicon Tandem Solar Cell on 16 $cm^2$. *ACS Energy Lett.* **3**, 2299–2300 (2018).

19. Qiu, Z. *et al.* Monolithic perovskite/Si tandem solar cells exceeding 22% efficiency via optimizing top cell absorber. *Nano Energy* **53**, 798–807 (2018).

20. Wu, Y. *et al.* Monolithic perovskite/silicon-homojunction tandem solar cell with over 22% efficiency. *Energy Environ. Sci.* **10**, 2472–2479 (2017).

21. Sahli, F. *et al.* Improved Optics in Monolithic Perovskite/Silicon Tandem Solar Cells with a Nanocrystalline Silicon Recombination Junction. *Adv. Energy Mater.* **8**, 1701609 (2018).

22. Bush, K. A. *et al.* 23.6%-efficient monolithic perovskite/silicon tandem solar cells with improved stability. *Nat. Energy* **2**, 17009 (2017).

23. Shen, H. *et al.* In situ recombination junction between p-Si and $TiO_2$ enables high-efficiency monolithic perovskite/Si tandem cells. *Sci. Adv.* **4**, eaau9711 (2018).

24. Bush, K. A. *et al.* Minimizing Current and Voltage Losses to Reach 25% Efficient Monolithic Two-Terminal Perovskite–Silicon Tandem Solar Cells. *ACS Energy Lett.* **3**, 2173–2180 (2018).

25. Nogay, G. *et al.* 25.1%-Efficient Monolithic Perovskite/Silicon Tandem Solar Cell Based on a *p* -type Monocrystalline Textured Silicon Wafer and High-Temperature Passivating Contacts. *ACS Energy Lett.* **4**, 844–845 (2019).

26. Mazzarella, L. *et al.* Infrared Light Management Using a Nanocrystalline Silicon Oxide Interlayer in Monolithic Perovskite/Silicon Heterojunction Tandem Solar Cells with Efficiency above 25%. *Adv. Energy Mater.* **9**, 1803241 (2019).

27. Sahli, F. *et al.* Fully textured monolithic perovskite/silicon tandem solar cells with 25.2% power conversion efficiency. *Nat. Mater.* **17**, 820–826 (2018).

28. Chen, B. *et al.* Grain Engineering for Perovskite/Silicon Monolithic Tandem Solar Cells with Efficiency of 25.4%. *Joule* **3**, 177–190 (2019).

29. Jošt, M. *et al.* Textured interfaces in monolithic perovskite/silicon tandem solar cells: advanced light management for improved efficiency and energy yield. *Energy Environ. Sci.* **11**, 3511–3523 (2018).

30. Köhnen, E. *et al.* Highly efficient monolithic perovskite silicon tandem solar cells: analyzing the influence of current mismatch on device performance. *Sustain. Energy Fuels* **3**, 1995–2005 (2019).
