# Supplementary material for: Enhanced optical path and electron diffusion length enable high-efficiency perovskite tandems
Source: Nat Commun. 2020 Mar 9;11:1257. doi: 10.1038/s41467-020-15077-3 (PMC7062737; doi:10.1038/s41467-020-15077-3)
Supplement: Supplementary file 3 — Reporting Summary [file 41467_2020_15077_MOESM3_ESM.pdf]

nature research

Corresponding author(s): Edward H. Sargent

# Solar Cells Reporting Summary

Nature Research wishes to improve the reproducibility of the work that we publish. This form is intended for publication with all accepted papers reporting the characterization of photovoltaic devices and provides structure for consistency and transparency in reporting. Some list items might not apply to an individual manuscript, but all fields must be completed for clarity.

For further information on Nature Research policies, including our data availability policy, see Authors & Referees.

## ü Experimental design

**Please check: are the following details reported in the manuscript?**

### 1. Dimensions

| Item | Yes/No | Details |
|---|---|---|
| Area of the tested solar cells | ☒ Yes ☐ No | The small area of solar cells is 0.053 cm2, and large area device is 1.95 cm2 (Methods) |
| Method used to determine the device area | ☒ Yes ☐ No | The active area is 0.049 cm2, 0.49 cm2, 1 cm2 and 1.68 cm2, determined by the aperture shade mask (Methods) |

### 2. Current-voltage characterization

| Item | Yes/No | Details |
|---|---|---|
| Current density-voltage (J-V) plots in both forward and backward direction | ☒ Yes ☐ No | Supplementary Figure 4b |
| Voltage scan conditions<br>*For instance: scan direction, speed, dwell times* | ☒ Yes ☐ No | JV curves were measured with a scanning rate of 100 mV/s (voltage step of 10 mV and delay time of 100 ms) (Methods, device haracterization) |
| Test environment<br>*For instance: characterization temperature, in air or in glove box* | ☒ Yes ☐ No | Performance measurements were carried in nitrogen environment (Methods) |
| Protocol for preconditioning of the device before its characterization | ☒ Yes ☐ No | No preconditioning was used |
| Stability of the J-V characteristic<br>*Verified with time evolution of the maximum power point or with the photocurrent at maximum power point; see ref. 7 for details.* | ☒ Yes ☐ No | Maximum power point tracking (Supplementary Figure 4c and d) |

### 3. Hysteresis or any other unusual behaviour

| Item | Yes/No | Details |
|---|---|---|
| Description of the unusual behaviour observed during the characterization | ☒ Yes ☐ No | Optimized cells showed negligible hysteresis |
| Related experimental data | ☒ Yes ☐ No | Supplementary Figure 4 |

### 4. Efficiency

| Item | Yes/No | Details |
|---|---|---|
| External quantum efficiency (EQE) or incident photons to current efficiency (IPCE) | ☒ Yes ☐ No | Figure 3 and Figure 6 |
| A comparison between the integrated response under the standard reference spectrum and the response measure under the simulator | ☒ Yes ☐ No | Figure 3 and Figure 6 |
| For tandem solar cells, the bias illumination and bias voltage used for each subcell | ☒ Yes ☐ No | This work is for 4T tandem (device characterizations) |

### 5. Calibration

| Item | Yes/No | Details |
|---|---|---|
| Light source and reference cell or sensor used for the characterization | ☒ Yes ☐ No | Newport, Class A simulator is used for the measurements (Methods, device characterization) |
| Confirmation that the reference cell was calibrated and certified | ☒ Yes ☐ No | The light intensity was calibrated by reference solar cell by Newport |

Calculation of spectral mismatch between the reference cell and the devices under test

- [x] Yes
- [ ] No

Mismatch factor of 1 was used in our measurements

6. Mask/aperture

Size of the mask/aperture used during testing

- [x] Yes
- [ ] No

0.049, 0.49, 1 and 1.68 cm2 (Methods, device characterization)

Variation of the measured short-circuit current density with the mask/aperture area

- [x] Yes
- [ ] No

Supplementary Figure 8

7. Performance certification

Identity of the independent certification laboratory that confirmed the photovoltaic performance

- [ ] Yes
- [x] No

We did not certify our cells. But CsMAFA control devices were certified and reported in our previous paper (Science 2017, 355, 722)

A copy of any certificate(s)
*Provide in Supplementary Information*

- [ ] Yes
- [x] No

We did not certify our cells

8. Statistics

Number of solar cells tested

- [x] Yes
- [ ] No

At least 20 devices for each composition were tested (Supplementary Figure 4a)

Statistical analysis of the device performance

- [x] Yes
- [ ] No

Supplementary Figure 4

9. Long-term stability analysis

Type of analysis, bias conditions and environmental conditions
*For instance: illumination type, temperature, atmosphere humidity, encapsulation method, preconditioning temperature*

- [x] Yes
- [ ] No

The stability test at MPP operation conditions under AM 1.5G simulated illumination with a 420-nm cutoff UV-filter was carried out in nitrogen for unencapsulated solar cells (Methods, Supporting information S5)
